# Susceptibility to Nocebo Hyperalgesia, Dispositional Optimism, and Trait Anxiety as Predictors of Nocebo Hyperalgesia Reduction

**DOI:** 10.1097/AJP.0000000000001112

**Published:** 2023-04-17

**Authors:** Merve Karacaoglu, Simone Meijer, Kaya J. Peerdeman, Elise Dusseldorp, Karin B. Jensen, Dieuwke S. Veldhuijzen, Henriët van Middendorp, Andrea W.M. Evers

**Affiliations:** * Health, Medical and Neuropsychology Unit; ‡ Methodology & Statistics Unit, Leiden University; † Leiden Institute for Brain and Cognition (LIBC); ∥ Department of Psychiatry, Leiden University Medical Center, Leiden; ¶ Medical Delta, Erasmus University Rotterdam, Leiden University & Delft University of Technology, Rotterdam/Leiden/Delft, The Netherlands; § Department of Clinical Neuroscience, Karolinska Institute, Stockholm, Sweden

**Keywords:** Nocebo effect, pressure pain, hyperalgesia, prediction, counterconditioning

## Abstract

The current paper explores the psychological predictors of nocebo hyperalgesia and whether the reduction of nocebo hyperalgesia can be predicted by susceptibility to nocebo hyperalgesia and psychological characteristics.

**Methods:**

Nocebo effects on pressure pain were first experimentally induced in 83 healthy female participants through conditioning with open-label instructions about the pain-worsening function of a sham TENS device to assess susceptibility to nocebo hyperalgesia. Participants were then randomized to 1 out of 2 nocebo-reduction conditions (counterconditioning/extinction) or to continued nocebo-conditioning (control), each combined with open-label instructions about the new sham device function. Dispositional optimism, trait and state anxiety, pain catastrophizing, fear of pain, and body vigilance were assessed at baseline.

**Results:**

The results showed that lower optimism and higher trait anxiety were related to a stronger induction of nocebo hyperalgesia. Moreover, a stronger induction of nocebo hyperalgesia and higher trait anxiety predicted a larger nocebo reduction across interventions. Also, nocebo hyperalgesia and optimism moderated the effects of the nocebo-reduction interventions, whereby larger nocebo hyperalgesia and lower optimism were associated with a larger nocebo reduction after counterconditioning, compared with control, and also extinction for larger nocebo hyperalgesia.

**Discussion:**

Our findings suggest that open-label conditioning leads to stronger nocebo hyperalgesia when trait anxiety is high and dispositional optimism is low, while these psychological characteristics, along with larger nocebo hyperalgesia, also predict open-label counterconditioning to be an effective nocebo-reduction strategy. Susceptibility to nocebo hyperalgesia, trait anxiety, and dispositional optimism might be indicators of a flexible pain regulatory system.

Nocebo effects are adverse treatment outcomes that are not attributable to active treatment components.^1^ They can be induced through learning processes of classic conditioning and instructional learning.^2^ Recently, studies have investigated the learning processes for reducing nocebo effects.^3^–^6^ Among these, extinction works by no longer reinforcing,^7^ or in other words no longer strengthening, the association between pain increase and a (sham) treatment, whereas counterconditioning is a method actively targeting the reversal of painful associations with a (sham) treatment. Findings suggest that counterconditioning is a more successful method for reducing nocebo hyperalgesia than extinction.^3^,^5^ However, research is still lacking on which individual differences predict susceptibility to nocebo effects or, equally importantly, the recovery therefrom.

Individuals differ in the degree to which they are susceptible to learning negative associations that result in nocebo effects.^2^,^8^ Here, susceptibility is a continuous term referring to the tendency to being influenced by experimental manipulation or a psychological characteristic. Research into psychological characteristics provides some indications for people high on fear or anxiety and low on optimism to be more susceptible to nocebo effects, while other research shows no associations for these or shows even weaker evidence for other expectancy-related traits, such as for higher pain catastrophizing or body vigilance.^9^–^11^ Individual differences may also exist during nocebo reduction. Research is needed to examine whether psychological characteristics and susceptibility towards nocebo hyperalgesia predict the level of nocebo reduction by different learning interventions. Possibly, a larger baseline nocebo hyperalgesia could be associated with more resistance to nocebo reduction,^12^,^13^ although the opposite might be true if larger nocebo hyperalgesia leads to a stronger desire for pain relief, which might increase the intervention efficacy.^14^

To this end, the current research entails additional exploratory analyses on a study in which the open-label induction and reduction of nocebo effects on pressure pain were investigated in a healthy female sample.^5^ Adding onto their findings, the current research aims are 4-fold. First, we explore whether any of the 6 psychological characteristics namely, dispositional optimism, state and trait anxiety, pain catastrophizing, fear of pain, and body vigilance, predict the strength of nocebo hyperalgesia after conditioning with open-label instructions about the pain-increasing function of a sham Transcutaneous Electrical Nerve Stimulation (TENS) device. Second and third, we investigate the predictive roles of susceptibility to the induction of nocebo hyperalgesia and psychological characteristics in the magnitude of nocebo change after 2 nocebo-reduction interventions, that is, counterconditioning and extinction combined with open-label instructions, with continued open-label nocebo conditioning serving as a control condition. Fourth, we explore whether susceptibility to the induction of nocebo hyperalgesia and psychological characteristics moderate the effects of these nocebo-reduction interventions. This extensive exploration of the predictors of nocebo reduction is novel and can be useful in the future for selecting the most effective nocebo-reduction strategy (either counterconditioning or extinction) based on individual differences.

## METHODS

### Design

**FIGURE 1:**
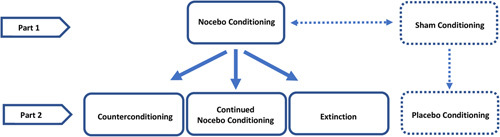
Overview of the full study design. Solid lines indicate the experimental groups that are part of the current study.

The current research is part of a larger study^5^ approved by the Psychology Research Ethics Committee of Leiden University (CEP18-1114/442; pre-registration ICTRP Trial ID: NL8033). In line with the aims of the current research, only a subset of experimental conditions from the larger study was considered for analysis, which entailed the manipulations for inducing and reducing nocebo effects on pressure pain (Fig. 1). For further details on all experimental conditions, including the larger study aims and their findings, the readers are referred to a separate publication.^5^ Data was used from the same sample, which has a sufficient sample size for conducting the planned analyses of the current research.^15^ During the experiment, participants were randomly allocated to a condition where nocebo effects were induced (nocebo conditioning) and subsequently, they were further allocated (1:1:1) to either 1 of the 2 nocebo-reduction conditions, counterconditioning or extinction, or to the control group, continued nocebo conditioning. The idea behind this 2-step design was to create an experimental model that potentially mimics real-life learning events where nocebo effects are induced and then altered by various learning processes. Moreover, in all groups, open-label instructions were provided about the function of a sham TENS device. Open-label instructions were chosen to allow for more ethical implementation of this design as a possible nocebo-reduction strategy for future clinical practice. Findings from open-label placebo studies indicate that an inert treatment can be prescribed without the concealment of their non-pharmacological contents, that is, without deception.^16^ Positive treatment outcomes can still be achieved by combining placebo administration with the rationale that placebo mechanisms can lead to the medical improvement of symptoms.^16^–^18^ The current experiment applied this open-label rationale to induce and reduce nocebo effects by using a sham TENS device as the inert treatment. As such, participants were informed about the inefficacy of the sham TENS device, that is, in reality, it cannot send electrical signals, but that through expectation mechanisms, the sham activation of the device can lead to either pain in- or a decrease in line with the instructions given about the device.

### Participants

Healthy females between 18 and 35 years with a good understanding of the Dutch language were recruited for the study. Since pressure pain is an ecologically valid stimulus type for disorders involving musculoskeletal pain,^19^,^20^ in which nocebo effects may play a clinically relevant role, and because adult women report more musculoskeletal problems than men,^21^ our sample consisted of only female participants to increase the generalizability of current findings to the clinical studies involving pressure pain. The exclusion criteria were: severe physical or psychological disorders, chronic pain complaints (≥3 mo) in the past or present, pain on the day of the experiment, injuries on the hands, Reynaud’s disease, color-blindness, pregnancy or breastfeeding, and current use of medication except for contraceptives. Participants were asked not to drink alcohol and not to use any analgesics, sleep medication, or recreational drugs within the 24 hours before the experiment. They were also asked not to wear any nail polish or acrylic nails on the thumb nail of their nondominant hand. An exclusion criterion during the first phase of the lab session, the pain calibration phase, was the inability to reliably distinguish between different pressure intensities.

Participants were recruited through posters and flyers distributed and handed out at various locations within Leiden University and through the online participant recruitment platform Sona (Sona Systems, Tallinn, Estonia). The study consisted of a single experimental session of 2 hours, which took place in the Faculty of Social and Behavioral Sciences labs of Leiden University. Participants were compensated with either cash (€15) or study credits for their participation.

### Pressure Pain Application

Pressure pain was induced on the thumbnail using a custom-made automated, pneumatic, computer-controlled pressure administrator, which was borrowed from the Karolinska Institute in Sweden,^22^ including a hand-piece borrowed from King’s College London. This device is still investigational. The thumb of the nondominant hand was inserted into the transparent handpiece, which applied pressure to the middle of the thumb nail through a piston with a 1 cm^2^ probe. Each stimulus lasted 2.5 seconds, with a 30 second inter-stimulus interval. The device could only maximally apply 850 kPa (≅8.7 kgf/cm^2^) pressure on the thumb nail, which is an intensity lower than the average that can be tolerated in healthy participants^23^ and was chosen as a safety measure considering the repetitive stimulus administration. In addition, an emergency stop button was provided so that participants could stop the pressure stimuli at any given moment during the experiment if they could not endure the pressure.

#### Pain Measurement

Participants verbally rated the pain intensity of each pressure stimulus on a Numeric Rating Scale (NRS), with the endpoints 0 representing no pain and 10 worst pain imaginable. Participants were able to rate their pain up to a decimal point. They were asked to only rate above zero (thus 0.1 and upwards) when they started to feel pain next to feeling a sensation of pressure. The verbally reported NRS ratings were entered into a computer by the experimenter after each trial.

#### Pressure Pain Calibration

Pressure pain was individually calibrated to evoke similar pain levels across participants due to expected individual differences in sensitization.^24^ Pressure intensities starting from 100 kPA (≅1 kgf/cm^2^) were administered with 50 kPA (≅0.5 kgf/cm^2^) increments on the thumb nail until participants rated ≥5.5 on the NRS or until 850 kPA (≅8.7 kgf/cm^2^) was reached. Based on the highest intensity of pressure scored as zero on the NRS and the highest scored pressure intensity, 3 new intermittent intensities were calculated that were equidistant from each other in magnitude. Together, these 5 intensities were randomly administered 3 times to determine the pressure intensities rated between the ranges 0 to 1, 2 to 3, and 4.5 to 5.5 on the NRS to determine nonpainful, slightly painful, and moderately painful pressure intensities, respectively. Since participants were allowed to rate using decimal points on the NRS, a barely painful pressure intensity (0–1 on the NRS) was also accepted as nonpainful, as it was expected that the repeated administration of pressure stimuli could lead to a slight sensitization, which could increase the nonpainful rating higher than zero. When the participants did not rate within the targeted range, standard formulas were used to interpolate the mid-value of the target range based on surrounding ratings.^5^ A calibration check followed where the pressure stimuli for nonpainful, slightly painful, and moderately painful intensities were randomly administered with slightly painful pressure intensity presented thrice and the rest presented twice. The pressure intensities were adjusted based on the same formulas if they were rated more than once outside of the target range. Five-minute breaks were taken between each calibration step to minimize stimulus sensitization. Breaks were extended by 1 minute, up to 5 minutes, if the participant indicated having pain ≥2 on the NRS. No participants asked for a break longer than 5 minutes.

#### Sham TENS Device

A sham TENS device (BeurerEM80, Beurer GmbH, Ulm, Germany) was used for the conditioning paradigm, which was renamed as a Dermal Nerve Stimulation (DNS) device to avoid possible preconceptions about TENS from interfering with experimental manipulations. Two TENS electrodes were attached vertically on the radial side of the forearm of the nondominant hand. The device itself was switched on as seeing the light would suggest its activation to the participant, but actually, it was never activated; thus, electrical signals were not delivered at any stage of the experiment. This device is not labelled for use under discussion. E-prime version 3.0 (Psychology Software Tools, Pittsburg, PA) was used for presenting the texts “DNS on” and “DNS off” on a monitor screen to indicate the (sham) activation of the DNS device. These texts were presented in purple and yellow, counter-balanced across participants.

### Induction and Reduction of Nocebo Hyperalgesia

#### Nocebo Induction: Nocebo Conditioning in Part 1

All participants were given open-label instructions about nocebo effects and how they can be induced by the principles of classic conditioning. They were informed that a sham nerve stimulator, called DNS, would be used for conditioning them to expect pain increase. This would be achieved by administering a moderately painful pressure stimulus to the thumb nail during the sham activation of the DNS device, but in fact, the DNS device would not send any electrical signals. The electrodes would remain attached to the arm to mimic the administration of electrical signals, similar to the act of swallowing a placebo pill even when knowing it does not contain any active components. During the 20 trials of the learning phase, participants were conditioned to expect a pain increase in half of the trials, hereafter referred to as experimental trials. For this, the text “DNS on” appeared on the screen 1 second before receiving a moderately painful pressure stimulus, and the experimenter pressed a button on the DNS to evoke a beep sound to indicate its sham activation. In the other half of the trials, that is, the control trials, “DNS off” appeared on the screen, and a slightly painful pressure stimulus was administered. Directly after the learning phase trials, the testing phase followed. During the testing phase, 3 trials were associated with “DNS on” and 3 trials with “DNS off”, where this time, both DNS conditions were paired with only slightly painful pressure stimuli. Participants were not informed during the open-label instructions that they would be receiving only slightly painful pressure stimuli during the testing phase.

#### Nocebo Reduction: Counterconditioning in Part 2

Participants allocated to the counterconditioning group were instructed that during this part of the experiment, they would be conditioned to expect to receive no pain instead of moderate pain when the DNS device is activated. This would be achieved by administering a nonpainful pressure stimulus during the sham activation of the device. The device (de)activation procedure was similar to part 1; except that this time a nonpainful pressure stimulus, instead of a moderately painful one, was paired with the text “DNS on” during half of the 20 learning trials and with a slightly painful pressure stimulus during the other half. During all 6 testing phase trials, only slightly painful pressure stimuli were administered unbeknownst to the participants regardless of DNS activation in half of the testing trials.

#### Nocebo Reduction: Extinction in Part 2

In the extinction group, participants received the instruction that this time they would be taught to expect no relation between the sham (de)activation of the DNS device and the amount of pain they receive. Therefore, during the sham activation of the DNS device, moderately painful pressure stimuli would no longer be administered. During all 20 learning trials and 6 testing phase trials, in which DNS was (de)activated in half of the trials, participants received only slightly painful pressure stimuli.

#### Control Condition: Continued Nocebo Conditioning in Part 2

In the continued nocebo conditioning group, participants were told that this part would be exactly as before and that they would receive higher pain during the sham activation of the DNS device compared with its sham deactivation. Same as in nocebo conditioning, participants received a moderately painful pressure stimulus during the experimental trials and a slightly painful pressure stimulus in the control trials of the learning phase. Again, only slightly painful pressure stimuli were administered unbeknownst to the participants during the testing phase trials.

### Operationalization of Nocebo Hyperalgesia and Nocebo Change

Nocebo effects were measured by calculating the mean difference between the pain ratings in all 3 experimental trials (“DNS on”) and the pain ratings in all 3 control trials (“DNS off”) from the testing phase in part 1 or 2. Nocebo hyperalgesia refers to the magnitude of nocebo effects obtained after nocebo conditioning in part 1. Nocebo change refers to changes in the magnitude of nocebo effects between parts 1 and 2. To obtain this variable, the nocebo effects calculated in part 2 were subtracted from the nocebo effects in part 1. A larger positive score on this nocebo change variable indicates a larger reduction in nocebo hyperalgesia from part 1 to part 2.

### Questionnaires

#### Dispositional Optimism

Dispositional optimism is the extent to which an individual believes that future outcomes will be good or positive.^25^ Based on a systematic review, lower levels of optimism were relatively consistently related to stronger nocebo responses, whereas higher levels of optimism were relatively consistently related to stronger placebo responses.^10^ The Life-Orientation Test-Revised (LOT-R) was used for assessing dispositional optimism.^26^ LOT-R is a 10-item measure containing positive items such as “In uncertain times, I usually expect the best,” negative items such as “If something can go wrong for me, it will,” and filler items. Respondents rate each item on a 5-point scale from 0=Strongly disagree to 4=Strongly agree. To calculate the optimism score, 3 negatively worded items are reverse coded and added to the 3 positively worded items, resulting in a total score from 0 to 24, with higher scores indicating higher optimism.

#### Trait Anxiety

Trait anxiety is regarded as a relatively stable personality trait, indicating individual differences in the intensity and frequency of perceiving stressful situations as dangerous or threatening.^27^ (Trait) Anxiety has been repeatedly found to correlate with a stronger nocebo response.^10^,^11^,^28^ The trait scale of the State-Trait Anxiety Inventory (STAI-T) was used for assessing trait anxiety.^27^ The scale contains 20 items about how a person generally feels, such as “I feel pleasant” or “I feel nervous and restless.” Respondents rate each item on a 4-point scale, with the endpoints 1=Almost never and 4=Almost always. To calculate the trait anxiety score, positively phrased items are reverse coded, and then the sum score of all items is calculated. The scores range between 20 and 80 points, with higher scores indicating greater trait anxiety.

#### State Anxiety

The state scale of the State-Trait Anxiety Inventory short-form (STAI-S-6) was used for assessing state anxiety.^29^ STAI-S-6 is sensitive to changes in transitory anxiety and indicates raised levels of anxiety at a given moment.^27^ When pain increase is anticipated within an environment, the resulting anticipatory anxiety has been found to lead to nocebo hyperalgesia.^30^ The scale consists of 6 items, measuring how respondents feel “right now, at this moment” with items such as “I feel calm” or “I feel tense.” STAI-S-6 is rated on a 4-point scale, with the endpoints 1=Not at all and 4=Very much so. Positive items were reverse coded, and then the sum score of all items was calculated. For comparability with the full STAI-S, scores were adjusted to range between 20 and 80 points, with higher scores indicating greater state anxiety.

#### Pain Catastrophizing

Catastrophizing is defined as an exaggerated negative mental state brought on by actual or anticipated painful experiences.^31^ One study found that pain catastrophizing was highly correlated with stronger nocebo effects on pressure pain induced by verbal suggestions and observational learning.^32^ However, the same group failed to find this correlation in another study with socially induced nocebo effects on pressure pain.^33^ The Pain Catastrophizing Scale (PCS) was used for assessing pain catastrophizing.^31^ PCS is a multidimensional construct that measures rumination (e.g., “I can’t stop thinking about how much it hurts”), magnification (e.g., “I become afraid that the pain will get worse”), and helplessness (e.g., “I feel I can’t go on”). It consists of 13 items rated on a 5-point scale, with the endpoints 0 = Not at all and 4=All the time. The sum score of all items was calculated, ranging from 0 to 52, with higher scores indicating more pain-catastrophizing thoughts.

#### Fear of Pain

Fear of pain is related to the emotional reactions surrounding actual or anticipated pain, leading to avoidance behavior, which may be more disabling than actual pain.^34^ Especially higher fear of medical pain was found to mediate the increase in stress levels following a nocebo intervention, where higher stress levels were related to greater nocebo hyperalgesia.^35^ Also, fear induced in subjects high in fear of pain was found to abolish the positive effects of placebo analgesia.^36^ The Fear of Pain Questionnaire-III (FPQ-III) was used for assessing fear of pain.^37^ FPQ-III is a 30-item questionnaire measuring fear related to severe pain (e.g., “Being in an automobile accident”), minor pain (e.g., “Biting your tongue while eating”), and medical pain (e.g., “Receiving an injection in your arm”). The FPQ-III is scored on a 5-point scale, with the endpoints 1 = Not at all and 5= Extreme, and the sum score of all items was calculated, ranging from 30 to 150, with a higher score indicating greater fear of pain.

#### Body Vigilance

The Body Vigilance Scale (BVS) was used for assessing the tendency to attend to bodily sensations.^38^ One study showed that the level of body vigilance moderated the increase in symptoms after taking a placebo that participants believed to be an actual drug.^39^ The more participants focused on their symptoms, the more symptoms they reported. In contrast, another study found that increased attention to somatic symptoms reduced pain levels when pain expectancy was high.^40^ BVS consists of 4 main items. Three items assess the degree of attentional focus, perceived sensitivity to changes in bodily sensations, and the average amount of time spent attending to bodily sensations. The fourth item involves rating how much attention is directed to 15 separate sensations such as “heart palpitations” or “feeling detached from self”. Ratings were made on 0 to 10 scales with endpoints 0= Strongly disagree and 10= Strongly agree for items 1 and 2, 0= Never and 10= Always for item 3, 0= Never and 10= Very much for the sensation ratings in item 4. Ratings in the fourth item were averaged to get a single score, and afterward the sum score of all 4 items was calculated, ranging from 0 to 40, with higher scores indicating a greater focus on bodily sensations.

### Procedure

After arriving at the lab, participants received information about the experiment, after which they signed an informed consent form and were screened for inclusion and exclusion criteria. If eligible, they continued with the experimental steps, starting with filling in psychological questionnaires (Qualtrics, Provo, UT), followed by participating in all measurements involving pressure stimuli. Nonpainful, slightly painful, and moderately painful pressure intensities were individually calibrated using the pressure pain device. After a successful calibration procedure, when participants were able to differentiate between the 3 pressure intensities, the experimenter opened the randomization envelope to randomly allocate participants to their respective experimental conditions for parts 1 and 2. Sham electrodes of the DNS device were attached to the arm, and further information was provided about the procedural steps in part 1. Twenty learning phase trials and 6 testing phase trials from part 1 followed. After a 10-minute break, participants received further instructions about the procedural steps in part 2. Again, 20 learning phase trials and 6 testing phase trials from part 2 followed. After the end of the experiment, the electrodes were removed from the arm, and participants were asked to fill in exit questionnaires, which were reported elsewhere.^5^ Afterward, participants were debriefed and reimbursed for their participation.

### Statistical Analyses

All statistical analyses were conducted using the R,^41^ version 4.1.0. The normality of study variables was checked, and log transformations were performed for the nocebo hyperalgesia score (skewness=0.92) and nocebo change score (skewness=0.96) due to a moderate skewness towards the right.^42^ However, we did not find any impact of data transformation on study results; therefore, it was decided to only report the results from nontransformed data to ease the interpretation of findings. Variance inflation factor values of independent variables were screened for multicollinearity, and this was not detected as all variance inflation factor values were below 10.^43^ For the regression analyses, residual scatterplots were visually inspected for the assumptions of normality, linearity, and homoscedasticity, which were not violated. Also, no influential values were detected (Cook’s *D*< 0.5). A *P* value below 0.05 was considered statistically significant. To assess reliability, Cronbach’s alpha levels were calculated for the psychological scales. Cronbach’s alpha levels ranged from .77 (LOT-R) to .93 (BVS), corresponding to acceptable to excellent internal consistency.^44^

To answer the first research question of whether psychological characteristics were related to nocebo hyperalgesia, their univariate relationships were tested using Pearson correlation coefficients, and their multivariate relationships were tested using a multiple regression analysis, where the standardized scores from 6 psychological characteristics were entered to the model as predictors with nocebo hyperalgesia as the outcome variable. For the remaining research questions, a hierarchical regression analysis was conducted to assess the statistical contribution of each block of predictors to the nocebo change score. All continuous predictors were centered around the mean to facilitate the interpretation of interaction effects.^43^ The analyses were performed twice. The first time the group variable was dummy coded with the control condition (ie, continued nocebo conditioning) as reference group. The second time, the extinction group was taken as the reference group. This enabled all 3 groups to be compared with each other. All predictors were entered into the model in 4 steps according to a predetermined order. In step 1, dummy variables of the group were added using force entry. In step 2, nocebo hyperalgesia was force entered into the model to answer the second research question, to identify the added value of nocebo hyperalgesia in predicting nocebo change from these experimental groups. Note that by including nocebo hyperalgesia as a covariate, the estimated model effects became identical for all possible outcome measures, that is, “nocebo change score” versus “raw intervention score”, which additionally justifies our decision on choosing “nocebo change score” over the “raw intervention score” as the outcome measure for this model, to facilitate the interpretation of findings.^45^ In step 3, 6 psychological characteristics were force entered to answer the third research question, to identify the added value of psychological characteristics in predicting nocebo change across groups. For the fourth research question, we investigated whether nocebo hyperalgesia and psychological characteristics moderated the group effects on nocebo change. Therefore, in step 4, 2-way-interaction terms between the dummy variables of group and nocebo hyperalgesia as well as between the dummy variables of group and each of the 6 psychological characteristics were included. To check whether the block of predictor(s) added at each step significantly contributed to an increase in the explained variance, ANOVA comparisons were performed between the nested models created in each subsequent step (ie, global tests).

To interpret the findings, the model created in step 2 was used for answering the second research question since the global test of this model represents the effect of the single variable entered in that step. For answering the third and fourth research questions, the effect of the global tests of steps 3 and 4, respectively, represent the effect of a group of variables; therefore, to be able to interpret the individual variables and to increase the interpretability of the model, we relied on the final model created with stepwise selection. When these global tests for steps 3 and 4 are significant, applying stepwise selection becomes warranted.^46^ As the stepwise selection method, we applied forward selection based on the largest decrease in the Akaike Information Criterion (AIC) between 2 models.^46^ With this selection procedure, the terms within the complex model (ie, with all possible predictors) were stepwise added to a simple one to obtain the most parsimonious model. As such, all predictors were stepwise added by the program following an automatic selection procedure. This procedure preserves the principle of marginality. Lastly, interaction plots were created for variables with a significant interaction effect.

## RESULTS

A total of 166 participants enrolled in the study. Seven participants were excluded during screening for not fulfilling the inclusion criteria, 46 participants were excluded during the pain calibration phase for not being able to reach a moderate pain rating for the highest administered pressure intensity, 3 participants were excluded due to technical problems, and 2 after pressing the emergency stop button due to pain sensitization, yielding 108 eligible participants of which 83 participated in the nocebo conditioning group. Therefore, a total of 83 healthy female participants (Mean age: 20.46, SD: 2.17) were included in the final analysis. Among these, 27 were allocated to counterconditioning, 29 to extinction, and 27 to continued nocebo conditioning in part 2.

To summarize the relevant findings from the larger study,^5^ nocebo effects were successfully induced in part 1, with significantly larger nocebo effects after nocebo conditioning than sham conditioning (ie, control). In part 2, a larger reduction of nocebo effects was found after counterconditioning compared with extinction and continued nocebo conditioning (ie, control).

The current analyses showed that nocebo hyperalgesia (*M*=1.29, SD=0.95) ranged between −0.33 and 4.37 points, whereby 95.2% (*N*=79) of participants had a positive score, indicating they were nocebo responders. Regardless of nocebo responsiveness, all participants were included in further prediction analyses. The mean nocebo change score across all 3 groups of part 2 was 0.91 (SD=1.37), which ranged between −2.17, indicating an increase in nocebo effects, and 5.47 points, indicating a nocebo reduction between part 1 and part 2. The mean change score in the counterconditioning group was 1.98 (SD=1.5), in the extinction group 0.77 (SD=0.90), and in the continued nocebo conditioning group −0.01 (SD=0.90).

### Psychological Predictors of Nocebo Hyperalgesia

An overview of means, SD, and the intercorrelations between nocebo hyperalgesia and 6 psychological characteristics are displayed in Table 1. Testing for univariate relationships, more trait anxiety (Pearson *r*=0.28, *P*<0.01) and less optimism (Pearson *r*=−0.22, *P*<0.05) were associated with larger nocebo hyperalgesia. Next, to test their multivariate relationship, nocebo hyperalgesia was regressed on all psychological characteristics in a multiple regression analysis (Table 2). Taken together, psychological characteristics did not significantly explain the variance in nocebo hyperalgesia (*F*(6,76)=1.9, *R* ^2^=0.062, *P*=0.09).

**TABLE 1:** Means, SDs, and Intercorrelations of Nocebo Hyperalgesia and Psychological Characteristics (*N*=83)

Variable	M (SD)	2	3	4	5	6	7
1. Nocebo hyperalgesia	1.29 (0.95)	–0.22*	0.28†	0.21	0.03	–0.03	0.14
2. Optimism	16.08 (3.68)	—	–0.58‡	–0.22*	–0.25*	–0.17	–0.09
3. Trait anxiety	36.71 (6.75)	—	—	0.66‡	0.43‡	0.38‡	0.27*
4. State Anxiety	32.97 (9.80)	—	—	—	0.32†	0.27*	0.24*
5. Pain catastrophizing	13.48 (7.58)	—	—	—	—	0.51‡	0.54‡
6. Fear of pain	71.29 (16.18)	—	—	—	—	—	0.32†
7. Body vigilance	19.39 (6.96)	—	—	—	—	—	—

* *P*< 0.05.

† *P*< 0.01.

‡ *P*<0.001 (2-tailed).

**TABLE 2:** Summary of Multiple Regression Analysis for Psychological Characteristics Predicting Nocebo Hyperalgesia (*N*=83)

	Nocebo hyperalgesia	
Variable	β	*P*
Intercept	1.29	< 0.001
Optimism	−0.10	0.44
Trait anxiety	0.23	0.19
State anxiety	0.07	0.62
Pain catastrophizing	−0.14	0.32
Fear of pain	−0.13	0.27
Body vigilance	0.17	0.18
Full model	Adj. *R* ^*2*^ *=0.06*	—
	*F* (6, 76)=1.9, *P*=0.09	—

β is the standardized regression coefficient.

### Predictors of Nocebo Reduction

Table 3 displays an overview of the hierarchical regression steps entered for creating the nested models. The ANOVA comparisons of all nested models, that is, the global tests, differed statistically from each other, indicating that each block of predictor(s) significantly increased the explained variance of the full model. As these global tests were significant, the forward selection was applied to the final model to increase interpretability.

**TABLE 3:** Summary of Hierarchical Regression Steps, the Explained Variance, and the ANOVA Tests of the Increase in Explained Variance From One Step to the Other (*N*=83)

	Nocebo change			
Variable	*R* ^2^	∆ R^2^	F-statistic	*df*
Step 1	0.35	—	—	—
Group	—	—	—	—
Step 2	0.61	0.26	52.13*	(79.1)
Nocebo hyperalgesia	—	—	—	—
Step 3	0.70	0.09	3.76†	(73.6)
Optimism	—	—	—	—
Trait anxiety	—	—	—	—
State anxiety	—	—	—	—
Pain catastrophizing	—	—	—	—
Fear of pain	—	—	—	—
Body vigilance	—	—	—	—
Step 4	0.79	0.09	1.92‡	(59.14)
Group×nocebo hyperalgesia	—	—	—	—
Group×optimism	—	—	—	—
Group×trait anxiety	—	—	—	—
Group×state anxiety	—	—	—	—
Group×pain catastrophizing	—	—	—	—
Group×fear of pain	—	—	—	—
Group×body vigilance	—	—	—	—

Only the variables kept in the model after forward selection are presented in the final model. *R* ^*2*^: Explained variance; ∆ *R* ^*2*^: Change in explained variance from one step to the other; *F-statistic:* F-statistic from one step to the other; *df*: Degrees of freedom.

* *P* <0.001 (2-tailed).

† *P* <0.01.

‡ *P* <0.05.

In step 1, the group variable significantly explained 35% of the variance in the nocebo change score. In line with the primary findings of the larger study,^5^ groups differed in nocebo change, with counterconditioning showing a significantly higher nocebo change score, indicating an average larger reduction in nocebo hyperalgesia, compared with both extinction and the control group, and extinction showing a significantly larger reduction in nocebo hyperalgesia compared with the control group (Tables 4 and 5).

**TABLE 4:** Summary of Final Model After Forward Selection Predicting Nocebo Change with Continued *Nocebo* Conditioning as Reference Group (*N*=83)

	Nocebo change		
Variable	∆ R^2^	∆ AIC	B step
Step 1	0.35*	—	—
Intercept	—	—	−0.01
Group A vs. C	—	—	1.99*
Group B vs. C	—	—	0.78†
Step 2	0.26*	—	—
Nocebo hyperalgesia	—	—	0.73*
Step 3 and 4 (with forward selection)	0.15*	—	—
Intercept	—	—	−0.06
Group A vs. C	—	—	1.99*
Group B vs. C	—	—	0.82*
Nocebo hyperalgesia	—	—	0.14
Optimism	—	−42.78	0.13‡
Trait anxiety	—	−45.40	0.03†
Group A vs. C×Nocebo Hyperalgesia	—	−41.40	1.07*
Group B vs. C×Nocebo Hyperalgesia	—	—	0.55†
Group A vs. C×Optimism	—	−47.78	−0.14†
Group B vs. C×Optimism	—	—	−0.06
Final Model	*Adj. R* ^*2*^=0.73 *F*(73,9)=25.87*		

Only the variables kept in the model after forward selection are presented in step 3 and 4.

Group A: Counterconditioning, Group B: Extinction, Group C: Continued Nocebo Conditioning; ∆ *R* ^*2*^: Change in explained variance; ∆ *AIC*: Change in Akaike’s Information Criterion after selecting this variable into the model; *B step* is the unstandardized coefficient for this variable at given analysis.

* *P* <0.001 (2-tailed).

† *P* <0.05.

‡ *P* <0.01.

**TABLE 5:** Summary of Final Model After Forward Selection Predicting Nocebo Change With Extinction as Reference Group (*N*=83)

	Nocebo Change		
Variable	∆ R^2^	∆ AIC	B step
Step 1	0.35*	—	—
Intercept	—	—	0.77*
Group A vs. B	—	—	1.21*
Group C vs. B	—	—	−0.78†
Step 2	0.26*	—	—
Nocebo Hyperalgesia	—	—	0.73*
Step 3 and 4 (with forward selection)	0.15*	—	—
Intercept	—	—	0.76*
Group A vs. B	—	—	1.17*
Group C vs. B	—	—	−0.82*
Nocebo Hyperalgesia	—	—	0.69*
Optimism	—	−42.78	0.07‡
Trait Anxiety	—	−45.40	0.03†
Group A vs. B×Nocebo Hyperalgesia	—	−41.40	0.52†
Group C vs. B×Nocebo Hyperalgesia	—	—	−0.55†
Group A vs. B×Optimism	—	−47.78	−0.08‡
Group C vs. B×Optimism	—	—	0.06‡
Final Model	*Adj. R* ^*2*^=0.73 *F* (73,9)=25.87*		

Only the variables kept in the model after forward selection are presented in steps 3 and 4.

Group A: Counterconditioning, Group B: Extinction, Group C: Continued Nocebo Conditioning; ∆ *R* ^*2*^: Change in explained variance; ∆ *AIC*: Change in Akaike’s Information Criterion after selecting this variable into the model; *B* step is the unstandardized coefficient for this variable at given analysis.

* *P* < 0.001 (two-tailed).

† *P* < 0.05.

‡ *P* < 0.01.

In step 2, nocebo hyperalgesia significantly explained an additional 26% of the variance in the nocebo change score, where a larger induction of nocebo hyperalgesia was associated with a significantly larger nocebo reduction (*b*=0.73, SE=0.10, *t=7.22, P*<0.001). This indicates that those participants who were more susceptible to acquiring nocebo hyperalgesia in part 1 were also more susceptible to learning new associations related to nocebo reduction in part 2.

In step 3, the inclusion of psychological characteristics significantly explained an additional 9% of the variance in the nocebo change score, and in step 4, the inclusion of moderators significantly explained an additional 9% of the variance. Because multiple variables were entered in steps 3 and 4, their individual contribution was interpreted as part of the final model created with forward selection.

**FIGURE 2:**
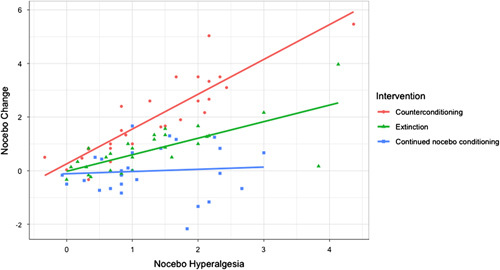
Nocebo hyperalgesia and intervention group as predictors of nocebo change. Note that higher levels on nocebo change indicate a larger reduction in nocebo effects from part 1 to part 2.

**FIGURE 3:**
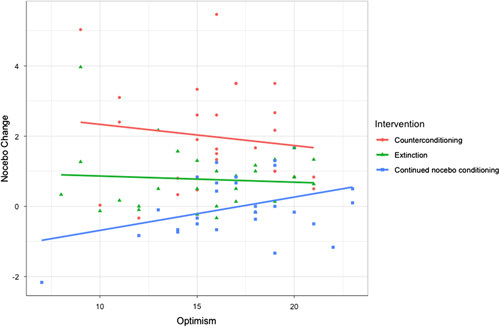
Optimism and intervention group as predictors of nocebo change. Note that higher levels on nocebo change indicate a larger reduction in nocebo effects from part 1 to part 2.

The forward selection resulted in the selection of group, nocebo hyperalgesia, optimism, trait anxiety, the interaction term of group and nocebo hyperalgesia, and the interaction term of group and optimism as the predictor variables in the final model (see Tables 4 and 5 for an overview). Together, the final model explained 73% of the variance in the nocebo change score. Trait anxiety was the only psychological characteristic with a significant main effect (*b*=0.03, SE=0.02, *t=2.08, P*=0.04) on nocebo change, whereby higher trait anxiety was associated with a larger nocebo reduction. Aside from this, there was a significant interaction between group and nocebo hyperalgesia. This interaction effect is plotted in Figure 2, where it can be observed that for lower levels of nocebo hyperalgesia, the type of intervention group does not strongly determine nocebo change, whereas for higher levels of nocebo hyperalgesia, counterconditioning results in a higher nocebo reduction than extinction, which in turn results in a higher nocebo reduction than continued nocebo conditioning. Moreover, there was a significant interaction of group and optimism on nocebo change for counterconditioning and continued nocebo conditioning groups. In line with the significant interaction effect between groups (A vs. C) and optimism (Table 4), it can be observed in Figure 3 that at lower levels of optimism, compared with higher optimism, the nocebo-reduction effect of counterconditioning was significantly larger compared with the continued nocebo conditioning group. Based on Figure 3, a similar trend holds for extinction compared with continued nocebo conditioning; however, this interaction effect was not statistically significant. Moreover, optimism levels did not moderate the intervention effect of counterconditioning compared with extinction.

## DISCUSSION

The current study investigated the predictors of nocebo hyperalgesia and nocebo change after interventions aimed at reducing nocebo hyperalgesia in a healthy female sample. This study entails additional exploratory analyses on a larger study,^5^ which can be useful for generating hypotheses for future research. Nocebo hyperalgesia was induced using experimental pressure pain by open-label conditioning and then reduced by open-label counterconditioning and open-label extinction, with continued open-label nocebo conditioning serving as the control group. The role of dispositional optimism, trait and state anxiety, pain catastrophizing, fear of pain, and body vigilance in the induction and reduction of nocebo hyperalgesia were explored. Their multivariate relationship with nocebo hyperalgesia was not significant; however, based on univariate relationships, higher trait anxiety, and lower optimism predicted larger nocebo hyperalgesia. Moreover, the main effects showed that larger nocebo hyperalgesia and higher trait anxiety predicted a larger nocebo reduction across groups. Interaction effects showed that for participants with larger nocebo hyperalgesia, compared with smaller, counterconditioning predicted a larger nocebo reduction than extinction and continued nocebo conditioning. For participants with lower optimism, compared with higher, counterconditioning was more effective than continued nocebo conditioning. Our findings provide initial indications that individual differences in nocebo hyperalgesia, as well as dispositional optimism and trait anxiety, could predict changes in nocebo hyperalgesia levels after nocebo-reduction interventions.

Investigation into the psychological differences indicated that higher trait anxiety and lower optimism predicted larger nocebo hyperalgesia. Only trait anxiety was a predictor of nocebo reduction across groups, which suggests that regardless of which nocebo-reduction strategy is selected, as trait anxiety increases not only the induction but also the reduction of nocebo hyperalgesia increases. This appears in contrast to a previous study, which found that higher levels of anxiety, measured by changes in autonomic arousal, perpetuate nocebo hyperalgesia and lead to resisting extinction.^47^ Speculatively, a potential explanation of our findings could be a heightened desire for pain relief experienced during high levels of anxiety, which could have facilitated the efficacy of the given intervention.^14^ Moreover, optimism moderated the intervention effects such that when optimism was low, compared with high, counterconditioning was more effective in reducing nocebo hyperalgesia compared with continued nocebo conditioning. It could be hypothesized that for pessimists, an intervention strategy might be more necessary than for optimists in reducing nocebo effects. Note as a limitation that a correction for multiple comparisons was not applied due to the exploratory nature of the current study. Although efforts to identify relevant psychological characteristics are still ongoing, a recent meta-analysis pointed towards consistent findings for the optimism-placebo and anxiety-nocebo associations across the literature,^10^ which is also largely in line with our current findings on nocebo hyperalgesia. Important to point out here is that the majority of existing studies in the field of placebo and nocebo research are closed-label, with only recent studies investigating less deceptive routes of placebo or nocebo administration.^16^ Among these, 1 open-label placebo study has looked into the role of personality characteristics in placebo response and found that optimism predicted the pain ratings in the deceptive placebo and no-treatment groups but not in the open-label placebo groups.^48^ Taken together, further research is recommended for investigating individual differences in open-label paradigms.

To the best of our knowledge, the current study is the first to suggest that susceptibility to nocebo hyperalgesia is an important predictor of nocebo reduction. A few studies have looked into the influence of prior experiences on subsequent nocebo and placebo effects.^12^,^13^ In these studies, participants’ positive or negative treatment expectations were first experimentally manipulated by either classic conditioning^12^,^13^ or observational learning,^13^ similar to how the current study induced nocebo hyperalgesia with (open-label) conditioning in part 1. Next, the carry-over effect of this manipulation was investigated for the pain ratings after the subsequent placebo or nocebo treatment. Their findings show that positive or negative prior learning experiences carry over to the placebo^12^,^13^ or nocebo response^13^ given to the subsequent treatment, respectively. Our findings, on the contrary, show that larger nocebo hyperalgesia predicts a larger nocebo reduction across interventions, although it should be noted that methodological differences exist between the current and previous studies. The current study quantified the amount of experimentally induced nocebo hyperalgesia, which was used as a predictor of nocebo intervention outcomes, instead of exploring the carry-over effects of nocebo hyperalgesia between interventions. This allowed us to determine whether nocebo-reduction strategies of counterconditioning and extinction are still effective^3^,^6^ when nocebo hyperalgesia is large. Our findings show that the effects of the more active reduction strategy, that is, counterconditioning compared with extinction, became stronger for individuals with larger nocebo hyperalgesia. A potential explanation of this finding could be that participants who are more susceptible to nocebo hyperalgesia might be susceptible to learning strategies in general, thereby responding equally strongly to the subsequent nocebo-reduction interventions. Also, the potential influence of ceiling or floor effects occurring in parts 1 and 2 cannot be ruled out entirely for their role in how much individual learning could actually take place during nocebo manipulations. Nevertheless, it seems nocebo hyperalgesia could be harnessed to strengthen the efficacy of nocebo-reduction interventions.

There are several clinical implications of our findings. In more than 95% of our healthy female sample, nocebo effects on pressure pain were successfully conditioned with an open-label suggestion. It is possible that in clinical populations, such as with chronic pain, the conditioning procedure results in more robust nocebo effects than in the healthy population. Potentially in chronic pain populations, increased exposure to negative treatment experiences and persistent pain could be associated with larger nocebo hyperalgesia^49^–^51^ than in healthy populations. Therefore, both the nocebo-induction and nocebo-reduction parts of our experiment should be investigated in clinical populations to make better inferences about the efficacy of open-label counterconditioning. Moreover, the current study identified a number of prognostic and prescriptive factors related to nocebo reduction. Prognostic factors are related to the general treatment outcomes regardless of treatment choice, whereas prescriptive factors predict individual differences in treatment response that can be used for deciding the most suitable treatment choice.^52^ Baseline trait anxiety was identified as a prognostic factor, whereas nocebo hyperalgesia and optimism levels were identified as prescriptive factors. Although open-label counterconditioning resulted in a larger overall mean change in nocebo hyperalgesia compared with other groups, and is therefore always recommended, it remains a good treatment choice especially when nocebo hyperalgesia is strong and when dispositional optimism is low. Moreover, individuals with higher trait anxiety are likely to benefit more than those with lower trait anxiety from any nocebo-reduction intervention; therefore, if treating a highly anxious individual, prescribing any 1 of the 2 interventions would likely result in nocebo reduction. Note that the current data is insufficient for making claims or recommendations about who would not benefit from these interventions. The generalizability of these findings should be further investigated in different clinical populations, in older populations, and also using sex/gender balanced designs for more specific treatment recommendations for nocebo reduction.

Several suggestions could be provided for future research directions. First, although the investigation of open-label treatment strategies is desirable due to ethical considerations,^53^ learning strategies such as conditioning and extinction likely do not occur as openly in daily life as we have introduced in this experiment. As a study limitation, our results may not be generalizable to daily life or be directly comparable with literature on closed-label paradigms. Future research is recommended to compare the efficacy of learning strategies in different contexts. Second, the nocebo training schedule in the current study was continuous, where the conditioned stimulus was consistently paired with the same pain intensity during the learning phase. In real-life, pain experiences are not consistently encountered in the same treatment contexts; therefore, it would be relevant to also test a more ecological variant of this learning model by including a partial reinforcement group to induce nocebo hyperalgesia and to test the efficacy of open-label counterconditioning also for this group. Third, it would be relevant to compare the efficacy of open-label counterconditioning to the nocebo-preventive strategies. Preliminary findings provide evidence for the efficacy of latent inhibition and overshadowing in inhibiting nocebo effects,^54^,^55^ while also contingency degradation is promising.^54^

## CONCLUSIONS

To conclude, lower optimism and higher trait anxiety predict larger nocebo hyperalgesia. Open-label counterconditioning appears to be an especially promising method for reducing (open-label) nocebo hyperalgesia in individuals who are highly susceptible to acquiring nocebo hyperalgesia. Moreover, individuals with high trait anxiety are likely to benefit from either counterconditioning or extinction, whereas for individuals with low optimism, counterconditioning, compared to control, is more effective. Our findings suggest that susceptibility to nocebo hyperalgesia, dispositional optimism, and trait anxiety might be indicators of a flexible pain-regulatory system that may shape pain experiences in both a negative and positive direction. Research into nocebo-reduction interventions could help personalize interventions to minimize nocebo effects in clinical practice.
